# Effects of CBD-Enriched *Cannabis sativa* Extract on Autism Spectrum Disorder Symptoms: An Observational Study of 18 Participants Undergoing Compassionate Use

**DOI:** 10.3389/fneur.2019.01145

**Published:** 2019-10-31

**Authors:** Paulo Fleury-Teixeira, Fabio Viegas Caixeta, Leandro Cruz Ramires da Silva, Joaquim Pereira Brasil-Neto, Renato Malcher-Lopes

**Affiliations:** ^1^ePrimeCare Healthcare SA, Belo Horizonte, Brazil; ^2^Department of Physiological Sciences, University of Brasilia, Brasilia, Brazil; ^3^Clinical Hospital, Federal University of Minas Gerais, Belo Horizonte, Brazil; ^4^Associação Brasileira de Pacientes de Cannabis Medicinal, Belo Horizonte, Brazil

**Keywords:** autism spectrum disorders, cannabidiol, epilepsy, *Cannabis sativa*, endocannabinoid system

## Abstract

Autism Spectrum Disorders comprise conditions that may affect cognitive development, motor skills, social interaction, communication, and behavior. This set of functional deficits often results in lack of independence for the diagnosed individuals, and severe distress for patients, families, and caregivers. There is a mounting body of evidence indicating the effectiveness of pure cannabidiol (CBD) and CBD-enriched *Cannabis sativa* extract (CE) for the treatment of autistic symptoms in refractory epilepsy patients. There is also increasing data support for the hypothesis that non-epileptic autism shares underlying etiological mechanisms with epilepsy. Here we report an observational study with a cohort of 18 autistic patients undergoing treatment with compassionate use of standardized CBD-enriched CE (with a CBD to THC ratio of 75/1). Among the 15 patients who adhered to the treatment (10 non-epileptic and five epileptic) only one patient showed lack of improvement in autistic symptoms. Due to adverse effects, three patients discontinued CE use before 1 month. After 6–9 months of treatment, most patients, including epileptic and non-epileptic, showed some level of improvement in more than one of the eight symptom categories evaluated: Attention Deficit/Hyperactivity Disorder; Behavioral Disorders; Motor Deficits; Autonomy Deficits; Communication and Social Interaction Deficits; Cognitive Deficits; Sleep Disorders and Seizures, with very infrequent and mild adverse effects. The strongest improvements were reported for Seizures, Attention Deficit/Hyperactivity Disorder, Sleep Disorders, and Communication and Social Interaction Deficits. This was especially true for the 10 non-epileptic patients, nine of which presented improvement equal to or above 30% in at least one of the eight categories, six presented improvement of 30% or more in at least two categories and four presented improvement equal to or above 30% in at least four symptom categories. Ten out of the 15 patients were using other medicines, and nine of these were able to keep the improvements even after reducing or withdrawing other medications. The results reported here are very promising and indicate that CBD-enriched CE may ameliorate multiple ASD symptoms even in non-epileptic patients, with substantial increase in life quality for both ASD patients and caretakers.

## Introduction

According to the DSM 5 (2013), Autism Spectrum Disorder (ASD) is characterized by functional deficits in three areas: mental development, social interaction, and behavior (1). In a multicenter epidemiological study done in 2012, involving nine countries, the median estimate of prevalence of ASD was 62/10.000 inhabitants (2). In clinical practice, the term ASD comprises a broad group of syndromes, diseases, and disorders (3, 4), that can affect cognitive development, motor skills, social interaction, communication, and behavior (frequently including auto and hetero-aggressiveness) (5–15). Often, this set of functional deficits results in incapacitation, lack of independence and severe distress for patients, families, and caregivers. For a recent review on this topic, refer to (16).

It is believed that ASD has multifactorial causes, generally associated with chromosomal or epigenetic changes in many different genes, which are often associated with neuronal function (17–24). Currently, there are no drugs or psychotherapeutic approaches capable of comprehensively improving life quality, social skills, and cognitive development of the most severe ASD patients (25–31). Currently available drugs may mitigate some specific symptoms, but generally speaking, they do so with a narrow range of effectiveness, and are often associated with important side effects (32, 33). Antipsychotic, antidepressant, or anxiolytic drugs, for example, may soothe autistic patients who display self-aggressive behavior (33–36). Antiepileptic drugs may be effective for seizure control and may even improve sleep quality and behavioral aspects (37). However, these drugs are known to cause major side effects (38–46). Moreover, none of these drus has been shown to significantly improve the lack of social interaction and communication skills that characterize and impose great impact on the lives of patients with ASD and their families.

Recent observational studies and trials reporting the use of pure CBD or CBD-enriched cannabis extracts for the treatment of syndromes characterized by refractory epilepsy and regressive autism suggest therapeutic potential of cannabinoids for autistic symptoms (47–60). These studies, which include extracts with a CBD/THC ratio of up to 20/1, showed that, even in children and adolescents, the side effects of these extracts are infrequent and less damaging than those reported for drugs traditionally used either for ASD, ADHD, sleep disorders, or epilepsy.

Changes in the expression of peripheral cannabinoid receptors were verified in autistic patients, suggesting possible deficiencies in the production and regulation of endogenous cannabinoids in ASD (61). This hypothesis has been recently confirmed specifically for anandamide, a major endocannabinoid, which is reduced in ASD patients (62). The understanding of the possible mechanisms involving the endocannabinoid system in the etiology of ASD has been derived from basic research in animal models. Special attention has been given to the neuronal hyperexcitability hypothesis and its possible relationship with the endocannabinoid system, which may also explain the higher incidence of epilepsy among ASD patients (63–68). Significant epileptform EEG activity has been recorded even in the central nervous system of non-epileptic autistic children (69), which is consistent with the “intense world hypothesis,” that relates autistic symptoms to excessive neuronal activity and connectivity (70). Together, these findings strongly support the need for testing *Cannabis sativa* extracts (CEs) and isolated phytocannabinoids as pharmacological approaches to control severe symptoms in both epileptic and non-epileptic ASD patients (68). Furthermore, CBD has been shown to have anxiolytic (71–75) and antipsychotic effects (76–79) in humans. It is plausible to assume that such effects are, at least in part, mediated by CBD-induced accumulation of the endocannabinoid anandamide (80). Although the mechanisms underlying CBD-induced antiepileptic effects are not entirely clear, anandamide modulation is likely to play an important role (68). In this context, it is interesting to note that anandamide accumulation, caused by inhibitors of its metabolic degradation, leads to reduction of social interaction deficits in the valproate-treated animal model of autism (81).

Here we report an observational study analyzing the effects of the compassionate use of *Cannabis sativa* extract (CE) containing a 75/1 CBD/THC ratio, which was given to a group of 18 ASD patients. The participant group includes 11 patients with no history of epilepsy, two previously diagnosed with epilepsy but seizure-free for over a year, and 5 currently diagnosed with epilepsy who had seizures during the month preceding treatment with CE. Treatment results were assessed by means of monthly questionnaires and clinical evaluation. The results after 6–9 months of treatment were extremely promising for both epileptic and non-epileptic patients. For the latter, observed improvements were much more comprehensive with fewer adverse effects than it would have been expected from currently available therapies. These preliminary results indicate, therefore, the urgent need for more extensive and detailed clinical studies to further validate the use of ECs and cannabinoids for the treatment of severe ASD symptoms.

## Materials and Methods

### Participants

The initial cohort included 18 ASD patients (ICD 10 = F84), aged 06–17 years (average 10), including five (28%) females and 13 (72%) males. Treatment with CE was spontaneously pursued by the patient's parents, who obtained legal authorization from the National Sanitary Surveillance Agency of Brazil (ANVISA) for the compassionate use of CE with all clinical assistance and treatment follow-up supervised by one of the authors of this article (P. F). Out of the 18 patients who had initiated treatment with standardized CE, three abandoned the treatment in the first month. Among the 15 patients who remained in the study, 05 had a diagnosis of epilepsy and had had seizures in the month preceding CE treatment, while the remaining 10 had never been diagnosed with epilepsy or had not had any clinical seizures for more than 12 months before treatment with CE. Among the five epileptic patients, one was diagnosed with Dravet's syndrome, two had epilepsy associated with cerebral palsy, and two had refractory epilepsy of undetermined etiology. Non-epileptic cases were randomly numbered 1–10, while epileptic cases were randomly numbered from 11 to 15. Demographic data are detailed in Table 1, while the individual patient's symptom profiles are detailed in Table 2.

**Table 1:** Cohort description and individual dosage^*^ of phytocannabinoids prescribed.

**Case #**	**Age (years)**	**Weight (kg)**	**CBD (mg/kg/day)**	**THC (mg/kg/day)**	**CBD (mg/day)**	**THC (mg/day)**
01^f^	07	25.0	4.00	0.05	100.00	1.33
02^m^	12	45.0	3.89	0.05	175.00	2.33
03^m^	09	33.0	3.79	0.05	125.00	1.67
04^m^	12	80.0	4.38	0.06	350.00	4.67
05^f^	11	34.0	5.88	0.08	200.00	2.67
06^m^	10	26.0	3.85	0.05	100.00	1.33
07^m^	09	32.0	3.91	0.05	125.00	1.67
08^f^	08	35.0	4.29	0.06	150.00	2.00
09^m^	14	49.0	4.08	0.05	200.00	2.67
10^m^	12	32.0	4.69	0.06	150.00	2.00
11^m^	18	89.5	3.35	0.04	300.00	4.00
12^m^	07	15.5	6.45	0.09	100.00	1.33
13^f^	15	46.0	5.43	0.07	250.00	3.33
14^m^	09	25.0	6.00	0.08	150.00	2.00
15^m^	11	35.0	4.29	0.06	150.00	2.00
Average	10.9	40.1	4.6	0.06	175.0	2.33
STD	3.06	20.18	0.94	0.01	74.40	0.99

* *The administration schedule was of two daily doses, one in the morning and one in the evening*.

f *Female patients*.

m *Male patients*.

**Table 2:** Caretakers' perception of improvement in each symptom category.

**Case #**	**Months of CE treatment^#^**	Perception of improvement of symptoms (%)^*^							
		**ADHD**	**BD**	**MD**	**AD**	**CSID**	**CD**	**SD**	SZ^**^
01^f^	09	15	15	-	10	15	15	50	-
02^m^	06	40	10	20	30	60	40	40	-
03^m^	09	40	30	40	20	40	30	50	-
04^m^	15	30	20	20	10	40	30	30	-
05^f^	27	50	25	35	20	25	35	40	-
06^m^	09	30	00	-	00	00	20	-	-
07^m^	09	15	15	15	15	15	15	50	-
08^f^	09	20	20	-	10	60	20	60	-
09^m^	09	00	−10	20	00	00	20	-	-
10^m^	09	30	25	20	10	30	15	-	-
11^m^	09	85	85	10	25	30	50	60	100
12^m^	09	50	00	55	00	40	10	25	≥50
13^f^	09	20	20	00	00	00	00	20	≥50
14^m^	39	35	40	20	15	25	30	85	≥50
15^m^	09	00	00	00	00	00	00	10	100
*n*^##^		15	15	12	15	15	15	12	05
Median		30	20	20	10	25	20	40	NA

*ADHD, Attention Deficit/Hyperactivity Disorder; BD, Behavioral Disorders; MD, Motor Deficits; AD, Autonomy Deficits; CSID, Communication and Social Interaction Deficits; CD, Cognitive Deficits; SD, Sleep Disorders; SZ, Seizures*.

f *female patients*.

m male patients.

* *Lack of improvement is computed as 00% and worsening of symptoms are recorded as negative values*.

# Total time of CE use, including before the onset of standardized CE.

## Number of patients presenting each symptom. A dash (-) indicate lack of the symptom before treatment onset. NA, Not applicable.

** *Scores for seizures are: 00, for lack of improvement, <50%, for reduction of <50% in the occurrence of SZ, ≥50%, for reduction of more than 50% in the occurrence of SZ; or 100% for cases of complete cessation of SZ*.

### Treatment

In August 2016, all patients started receiving standardized CE, with the same composition and origin, manufactured by CBDRx® (Colorado, USA). The standardized CE contained a proportion of ~75/1 CBD/THC and was administered orally in capsules containing 25 or 50 mg of CBD and ~0.34 or 0.68 mg of THC, respectively (according to data provided by the manufacturer).

From the 18 patients who started standardized CE treatment, 15 had never used any CE previously, while three had already used CEs for periods ranging from 5 to 24 months. The standardized CE doses were established individually by a titration process within a dose range based on CBD doses previously reported for use of CBD-enriched CEs for treatment of refractory epilepsy associated with regressive autism (53, 54, 57, 58, 60). Thus, the average initial dose of CBD was ~2.90 mg/kg/day, varying according to individual case severity at the beginning of treatment (minimum: 2.30 mg/kg/day and maximum: 3.60 mg/kg/day). Dosage adjustment was done intensively during the first 30 days and more sparsely over the following 150 days. The average dose of CBD administered from then until the end of the study was 4.55 mg/kg/day, with a minimum of 3.75 and a maximum of 6.45 mg/kg/day (Table 1). The average dose of THC in the same period was 0.06 mg/kg/day, with a minimum of 0.05 and a maximum of 0.09 mg/kg/day. Individual maintenance doses used by patients after the adjustment period are shown in Table 1, which does not include patients who abandoned the standardized CE treatment during the first month. The administration schedule was of two daily doses, one in the morning and one in the evening.

### Cannabinoid Extract Acquisition

By means of a non-commercial collaboration between the *Brazilian Association of Medicinal Cannabis Patients* (also known as AMAME) and the manufacturer CBDRx®, the standardized CE was donated by the company CBDRx LLC at no charge to the patients.

### Data Acquisition

The patient's parents and/or caregiver received a standardized form by e-mail (Supplementary Material), which should be answered once before the beginning of the study (baseline), and monthly throughout the duration of the CE treatment. In these forms the parents/caregivers were asked to estimate the severity of each of the eight symptom categories evaluated (see Supplementary Material). They should inform a score between 0 and 100, in which 0 means the lowest level of performance (or the maximum level of deficit and impairment associated to the symptom), and 100 means maximum performance (or complete absence of deficit and impairment associated to that symptom). The data presented here correspond to the difference observed between baseline results and results reported in the final month of treatment.

To ensure that the parents/caregiver properly understood the meaning of each category and that they were using the numeric scores in a consistent way throughout the study, the forms also contained two accessory questions (see Supplementary Material). In the first of these accessory questions the caregivers were asked to freely describe, in their own words, what changes they had observed since the last month. In the second accessory question parents/caregivers were asked to inform the degree of change in a 5-level Likert-like scale, for each group of symptoms, in relation to the previous month. The three different responses allowed the detection of inconsistencies. Every month the patient's physician (P. F.) checked the numeric evaluation for consistency, and whenever an inconsistency was observed the physician would contact the parent/caregiver, either in person or by phone, and ask them to consider adjusting the response.

### Evaluation of the Results

Patients were followed by means of periodic clinical evaluations made by the physician in charge. A monthly questionnaire was used to record treatment effects based on the answers given by the parents. Monthly standardized forms were filled out and contained questions covering the following symptom categories (see Supplementary Material for a detailed description of each category):

1. Attention Deficit/Hyperactivity Disorder (ADHD);
2. Behavioral Disorders (BD);
3. Motor Deficits (MD);
4. Autonomy Deficits (AD);
5. Communication and Social Interaction Deficits (CSID);
6. Cognitive Deficits (CD);
7. Sleep Disorders (SD);
8. Seizures (SZ).

Parents answered the initial questionnaires in August 2016 to assess the presence or absence of these symptoms before the onset of CE treatment. In the monthly questionnaires that followed for the next 9 months, until April 2017, the perceived percentage change for each symptom category was assessed. Clinical assessments and monthly records also included information regarding side effects and changes, maintenance, reduction, or withdrawal of neuropsychiatric drugs that were already in use (Table 2).

**Figure 1:**
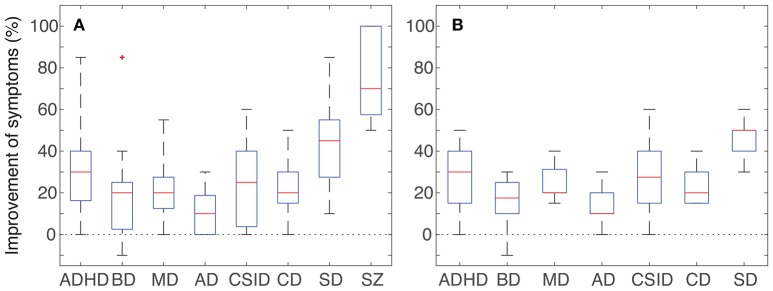
Improvement of symptoms observed in the patients that underwent CE treatment for at least 6 months. Data was collected from the caretakers' monthly follow-up questionnaires. **(A)** Pooled data from all 15 patients in the following categories: attention deficit/hyperactivity disorder (ADHD, *n* = 15); behavioral disorders (BD, *n* = 15); motor deficits (MD, *n* = 12); autonomy deficits (AD, *n* = 15); communication and social interaction deficits (CSID, *n* = 15); cognitive deficits (CD, *n* = 15); sleep disorders (SD, *n* = 12); convulsive seizures (SZ, *n* = 5). **(B)** Subset including only the 10 non-epileptic patients that underwent CE treatment for at least 6 months. Attention deficit/hyperactivity disorder (ADHD, *n* = 10); behavioral disorders (BD, *n* = 10); motor deficits (MD, *n* = 7); autonomy deficits (AD, *n* = 10); communication and social interaction deficits (CSID, *n* = 10); cognitive deficits (CD, *n* = 10); sleep disorders (SD, *n* = 7). Red lines denote medians, blue boxes the interquartile range, black whiskers the minimum and maximum values. Note that all categories present improvements that range from modest to robust, in spite of a very short period of treatment with CE.

The descriptive statistics in Figures 1A,B were plotted in MATLAB 2017a using the default settings of the *boxplot* function from the “Statistics and Machine Learning Toolbox.”

## Results

### General Results

Three patients (one female and two males, or 17% out of the cohort of 18 patients) chose to suspend treatment before the end of the first month due to the occurrence of adverse effects. In two of these patients a worsening of symptoms may have been due to the concomitant and unsupervised attempt to remove or reduce the dosage of antipsychotics. The third patient may have suffered adverse effects of the interaction of the prescribed cannabinoids with two other psychiatric medications that were being used simultaneously. For the remaining 15 patients that adhered to the standardized CE treatment, the consolidated results recorded during the final month of treatment are presented in Table 2 and graphically depicted in Figure 1A. Results for all non-epileptic patients are presented in Figure 1B. No differences were observed between genders, and for that reason results for both genders are shown together.

Overall, mostly positive outcomes were reported for the 15 patients that adhered to the standardized CE treatment (one case for 6 months and 14 cases for 9 months), especially regarding improvements in sleep disorders, seizures, and behavioral crisis. Also, signs of improvement were reported for motor development, communication and social interaction, and cognitive performance (Table 2). We highlight that 14 out of these 15 patients (93%) showed improvements equal to or above 30% in at least one symptom category. Most patients that adhered to the treatment had improvements in more than one symptom category: seven patients (47%) had improvements equal to or above 30% in four or more symptom categories; two patients (13%) presented improvements equal to or above 30% in two symptom categories, and five patients (33%) presented improvements equal to or above 30% in one symptom category. Only one patient, referred to as Case 9, who was receiving multiple neuropsychiatric medications throughout the study, presented overall maintenance or worsening of symptoms.

### Results Grouped by Symptom Categories

Clinical assessment and records of patients' evolution, which were filled in monthly by the patients' guardians/caretakers, targeted the main symptom categories associated with autism. Possible side effects of CE administration and modifications in the dosage of other neuropsychiatric drugs that were prescribed were also evaluated and are presented in Table 3. From the 15 patients who adhered to the treatment with standardized EC, 15 had symptoms of ADHD; 15 of BD; 12 of MD; 15 of AD; 15 of CSID; 15 of CD; 12 of SD; and 5 of SZ. Also, as shown in Table 3, 10 of these patients were also concomitantly taking other prescribed neuropsychiatric medications (OM).

**Table 3:** Neuropsychiatric drugs taken by each patient during the study.

**Case #**	**OM in use before CE treatment**	**OM in use after CE treatment**	**Summary of changes in OM**	**CE side effects**
01^f^	None	None	No OM	None
02^m^	Risperidone + Melatonin	None	Complete withdrawal	None
03^m^	None (used oxcarbazepine before the study)	None	No OM	None
04^m^	None	None	No OM	Nocturia and polyuria
05^f^	None (used several OM before the study)	None	Complete withdrawal	None
06^m^	None	None	No OM	Hyperaemia, sleepiness, and transient increase in core temperature
07^m^	Promethazine + Risperidone	Risperidone	Partial withdrawal	Transient sleepiness
08^f^	Melatonin + Risperidone	Risperidone	Partial withdrawal + dosage reduction	Slight increase in appetite
09^m^	Oxcarbazepine + Risperidone + Levomepromazine	Oxcarbazepine + Risperidone + Levomepromazine	None	None
10^m^	None	None	No OM	None
11^m^	Carbamazepine + Risperidone	Risperidone	Partial withdrawal + dosage reduction	Transient diarrhea at treatment onset
12^m^	Phenobarbital	Phenobarbital	Complete withdrawal	Nocturia
13^f^	Lamotrigine + Topiramate	Lamotrigine + Topiramate	Dosage reduction	Sleepiness and mild irritation
14^m^	Oxcarbazepine + Levetiracetam + Topiramate + Valproate semisodium + Risperidone	Topiramate + Risperidone	Partial withdrawal + dosage reduction	Transient sleepiness and mild irritation when waking up
15^m^	Risperidone + Oxcarbazepine	Risperidone + Oxcarbazepine	Dosage reduction	None

OM, alterations in other prescribed medication after introduction of CE (unaltered: no changes in the use of other medication was made; reduction, reduced the dosage of one or more medication; partial withdrawal, stopped completely the use of one of the medications; withdrawal, stopped completely the use of all other medication with the exception of CE.

f female patients;

m *male patients*.

At least 60% of patients showed improvements of 20% or more in ADHD, MD, CSID, BD, SD, and SZ. From the 15 patients who presented BD, eight (53.3%) had improvements equal to or above 20% in this symptom category. In AD, only four (26.7%) had improvements equal to or above 20%. The most robust results were found for ADHD, SD, and SZ, with more than 80% of patients presenting improvements equal to or above 30%. The results were particularly impressive for the control of seizures in the five epileptic patients, with seizure reduction of 50% in three cases and 100% in the other two cases. It is also worth noting that CE treatment made it possible to achieve a decrease in the dosage or to discontinue other neuropsychiatric medications in eight out of 10 patients that were receiving OM (Table 2).

### Untoward Effects

The following adverse effects were reported among the 15 patients who adhered to CE treatment: sleepiness, moderate irritability (three cases each); diarrhea, increased appetite, conjunctival hyperemia, and increased body temperature (one case each). All these side effects were mild and/or transient. Two patients presented nocturia, which in one case appeared concomitantly to an improvement in sleep quality.

As stated previously, three patients interrupted the treatment before the end of the first month of CE treatment due to adverse effects such as insomnia, irritability, increased heart rate, and worsening of psycho-behavioral crisis. Additionally, there was one patient (Case 2) who adhered to the treatment until the sixth month and, in spite of improvement in some respects, showed significant worsening in psycho-behavioral aspects. The patients who experienced relevant side effects were all receiving several drugs (Patient 1: Clomipramine + Pericyazine; Patient 2: Risperidone + Prometazine + Sodium Valproate; Patient 3: Risperidone + Prometazine), including at least one antipsychotic, and in two cases there was an abrupt cessation of the antipsychotic.

## Discussion

Here we report an observational study, which collected information provided by the clinician and the patients' parents during treatment of autistic patients with a CBD-enriched CE containing a rate of ~75:1 CBD to THC. Treatment duration ranged from 6 to 9 months. The initial cohort included 18 patients aged between 7 and 18. Three participants suspended CE use in the first 30 days of treatment, while 15 continued the use of standardized CE for six (01 patient) or nine (14 patients) months. All patients received the equivalent to an average CBD dose of 4.6 mg/kg/day and an average THC dose of 0.06 mg/kg/day. The prescribed THC dose is considered to be substantially below its safety margin (54). On the other hand, even low doses of pure THC, ranging from 0.04 to 0.12 mg/kg/day, have been previously shown to cause spasticity reduction, increased interest and connection with the environment, increased demonstration of initiative, reduction of seizure frequency, and improvement in dystonia of children with severe epileptic syndromes (82).

Previous studies have shown reliable efficacy and safety of CE containing a 20:1 CBD to THC proportion for the treatment of syndromes characterized by refractory epilepsy and regressive autism (54). Our positive results obtained from five epileptic patients (Table 1 and Figure 1A) corroborate the existing data regarding the effectiveness of CBD-enriched CE in the control of refractory seizures (47–60). Moreover, to the best of our knowledge, this is the first report of a marked improvement in autistic symptoms of non-epileptic patients with the use of CE (Figure 1B).

Not all patients benefited equally from the treatment. From the initial cohort of 18 patients, four patients reported negative results. All of these participants were receiving multiple drugs, including at least one antipsychotic, which suggests the occurrence of undesirable drug interactions. In one of these cases, we suspect that the worsening of symptoms may have been due to an abrupt and unsupervised withdrawal of an antipsychotic drug. These observations point to a potential risk of paradoxical effects when introducing CE in a drug combination that includes antipsychotic drugs. This underscores the need for extra vigilance and of a gradual increase in the dosage of EC in patients receiving many drugs, and also to evaluate with caution the possibility of either partial or complete withdrawal of previously prescribed drugs.

Among the 15 patients who adhered to treatment for at least 6 months, 10 were non-epileptic or had not experienced seizures for at least 1 year (Table 2 and Figure 1B). These patients showed positive effects in almost all evaluated categories, namely: ADHD, MD, AD, CSID, CD, and SD. Particularly among non-epileptic, nine (90%) presented improvement equal to or above 30% in at least one of these categories, six (60%) presented improvement of 30% or more in at least two categories, and four (40%) presented improvement equal to or above 30% in at least four symptom categories (Table 2). Therefore, the present observational study corroborates the notion that the range of therapeutic benefits of CBD-enriched CE extends to several distinct autistic symptoms, even in non-epileptic patients.

We note that due to the fact that the behavior/symptoms were annotated by caregivers, results on behavior improvement contain a significant degree of subjectivity. We also note that the reported results are subjectively quantitative, and that the degree of improvement may be non-linear (so that 60% improvement does not necessarily mean twice as much improvement as 30%).

Conspicuous positive effects, in both epileptic and non-epileptic patients, were observed in ADHD, SD, and CSID categories. It is evident that sleep quality improvement and hyperactivity reduction tend to have major positive impacts on mood and general health, as well as on the efficacy of psycho-pedagogic therapeutic interventions. Furthermore, in a long-term perspective, psycho-pedagogic therapy may potentiate the social, cognitive, and behavioral benefits observed after CE treatment. The least pronounced effects were seen on improvement of autonomy deficits (AD). This may indicate a need for a larger time interval to allow for consolidated routines and behavioral patterns, both from patients and from caretakers, to be remodeled before any benefit can be obtained from CE treatment.

The findings presented here, taken together, support the notion that many autism symptoms are associated to neuronal hyperexcitability, and indicate that CBD-enriched CE yields positive effects in multiple autistic symptoms, without causing the typical side effects found in medicated ASD patients. Most patients in this study had improved symptoms even after supervised weaning of other neuropsychiatric drugs. The intrinsic limitations of the present study, due to its observational nature, are the lack of control groups, the small cohort size, and potentially significant placebo effects (83). Further clinical trials are warranted to confirm these initial findings.

## Ethics Statement

The studies involving human participants were reviewed and approved by the Ethics Committee on Human Research of the Health Sciences College of the University of Brasilia (Universidade de Brasília- UnB), under the protocol number CAAE 16308719.3.0000.0030. Written informed consent to participate in this study was provided by the participants' legal guardian/next of kin.

## Author Contributions

PF-T: concept, methods design, patient care, clinical supervision, writing contributions to the manuscript introduction, methods, and discussion. FC: data analysis, critical review of the manuscript, submission. LR: concept, methods design, writing contributions to the manuscript introduction. JB-N: critical review of the manuscript. RM-L: concept, methods design, scientific supervision, bibliographic review, writing contributions to the manuscript introduction, methods, and discussion.

### Conflict of Interest

PF-T was employed by ePrimeCare Healthcare SA, Belo Horizonte, Brazil. RM-L has provided technical advisement to Grüne Labs, Pando, Uruguay. The remaining authors declare that the research was conducted in the absence of any commercial or financial relationships that could be construed as a potential conflict of interest.

## References

[1] American Psychiatry Association Diagnostic and Statistical Manual of Mental Disorders - DSM-5, 5th ed. AP Association Editor, Belo Horizonte (2013).

[2] Elsabbagh M Divan G Koh YJ Kim YS Kauchali S Marcin C. Global prevalence of autism and other pervasive developmental disorders. Autism Res. (2012) 5:160–79. 10.1002/aur.239 22495912 PMC3763210

[3] Committee on Children With Disabilities American academy of pediatrics: the pediatrician's role in the diagnosis and management of autistic spectrum disorder in children. Pediatrics. (2001) 107:1221–6. 10.1542/peds.107.5.1221 11331713

[4] Mahajan R Bernal MP Panzer R Whitaker A Roberts W Handen B. Clinical practice pathways for evaluation and medication choice for attention-deficit/hyperactivity disorder symptoms in autism spectrum disorders. Pediatrics. (2012) 130(Suppl 2S):125–38. 10.1542/peds.2012-0900J 23118243

[5] Kern JK Geier DA King PG Sykes LK Mehta JA Geier MR. Shared brain connectivity issues, symptoms, and comorbidities in autism spectrum disorder, attention deficit/hyperactivity disorder, and tourette syndrome. Brain Connect. (2015) 5:321–35. 10.1089/brain.2014.0324 25602622

[6] Shuster J Perry A Bebko J Toplak ME. Review of factor analytic studies examining symptoms of autism spectrum disorders. J Autism Dev Disord. (2014) 44:90–110. 10.1007/s10803-013-1854-3 23729334

[7] Hazen EP Stornelli JL O'Rourke JA Koesterer K McDougle CJ. Sensory symptoms in autism spectrum disorders. Harv Rev Psychiatry. (2014) 22:112–24. 10.1097/01.HRP.0000445143.08773.58 24614766

[8] Kral TV Eriksen WT Souders MC Pinto-Martin JA. Eating behaviors, diet quality, and gastrointestinal symptoms in children with autism spectrum disorders: a brief review. J Pediatr Nurs. (2013) 28:548–56. 10.1016/j.pedn.2013.01.008 23531467

[9] Angelidou A Alysandratos KD Asadi S Zhang B Francis K Vasiadi M. Brief report: “allergic symptoms” in children with autism spectrum disorders. More than meets the eye? J Autism Dev Disord. (2011) 41:1579–85. 10.1007/s10803-010-1171-z 21210299

[10] Stoppelbein L Sytsma-Jordan S Greening L. Correlates of psychomotor symptoms in autism. Int Rev Neurobiol. (2005) 71:343–57. 10.1016/S0074-7742(05)71014-X 16512357

[11] Bozzi Y Provenzano G Casarosa S. Neurobiological bases of autism-epilepsy comorbidity: a focus on excitation/inhibition imbalance. Eur J Neurosci. (2018) 47:534–48. 10.1111/ejn.13595 28452083

[12] Cawthorpe D. Comprehensive description of comorbidity for autism spectrum disorder in a general population. Perm J. (2017) 21:16-088. 10.7812/TPP/16-088 28241914 PMC5283790

[13] Pan PY Yeh CB. The comorbidity of disruptive mood dysregulation disorder in autism spectrum disorder. Psychiatry Res. (2016) 241:108–09. 10.1016/j.psychres.2016.05.001 27161986

[14] Polyak A Kubina RM Girirajan S. Comorbidity of intellectual disability confounds ascertainment of autism: implications for genetic diagnosis. Am J Med Genet B Neuropsychiatr Genet. (2015) 168:600–8. 10.1002/ajmg.b.32338 26198689

[15] Stadnick N Chlebowski C Baker-Ericzen M Dyson M Garland A Brookman-Frazee L. Psychiatric comorbidity in autism spectrum disorder: correspondence between mental health clinician report and structured parent interview. Autism. (2017) 21:841–51. 10.1177/1362361316654083 27407039 PMC5226915

[16] Khalil R Tindle R Boraud T Moustafa AA Karim AA. Social decision making in autism: on the impact of mirror neurons, motor control, and imitative behaviors. CNS Neurosci Ther. (2018) 24:669–76. 10.1111/cns.13001 29963752 PMC6055683

[17] Griffiths KK Levy RJ. Evidence of mitochondrial dysfunction in autism: biochemical links, genetic-based associations, and non-energy-related mechanisms. Oxid Med Cell Longev. (2017) 2017:4314025. 10.1155/2017/4314025 28630658 PMC5467355

[18] Hu Y Ehli EA Boomsma DI. MicroRNAs as biomarkers for psychiatric disorders with a focus on autism spectrum disorder: current progress in genetic association studies, expression profiling, and translational research. Autism Res. (2017) 10:1184–203. 10.1002/aur.1789 28419777

[19] Schroeder JC Reim D Boeckers TM Schmeisser MJ. Genetic animal models for autism spectrum disorder. Curr Top Behav Neurosci. (2017) 30:311–24. 10.1007/7854_2015_407 26602248

[20] Todd RD. Genetic advances in autism hinge on the method of measuring symptoms. Curr Psychiatry Rep. (2005) 7:133–7. 10.1007/s11920-005-0010-y 15802090

[21] Tordjman S Cohen D Coulon N Anderson GM Botbol M Canitano R Reframing autism as a behavioral syndrome and not a specific mental disorder: implications of genetic and phenotypic heterogeneity. Neurosci Biobehav Rev. (2017) 80:210 10.1016/j.neubiorev.2018.01.014 28153685

[22] Ziats MN Rennert OM. The evolving diagnostic and genetic landscapes of autism spectrum disorder. Front Genet. (2016) 7:65. 10.3389/fgene.2016.00065 27200076 PMC4844926

[23] Trottier G Srivastava L Walker CD. Etiology of infantile autism: a review of recent advances in genetic and neurobiological research. J Psychiatry Neurosci. (1999) 24:103–15. 10212552 PMC1188990

[24] Walsh P Elsabbagh M Bolton P Singh I. In search of biomarkers for autism: scientific, social and ethical challenges. Nat Rev Neurosci. (2011) 12:603–12. 10.1038/nrn3113 21931335

[25] Masi A Lampit A DeMayo MM Glozier N Hickie IB Guastella AJ. A comprehensive systematic review and meta-analysis of pharmacological and dietary supplement interventions in paediatric autism: moderators of treatment response and recommendations for future research. Psychol Med. (2017) 47:1323–34. 10.1017/S0033291716003457 28091344

[26] Masi A DeMayo MM Glozier N Guastella AJ. An overview of autism spectrum disorder, heterogeneity and treatment options. Neurosci Bull. (2017) 33:183–93. 10.1007/s12264-017-0100-y 28213805 PMC5360849

[27] Lei J Ventola P. Pivotal response treatment for autism spectrum disorder: current perspectives. Neuropsychiatr Dis Treat. (2017) 13:1613–26. 10.2147/NDT.S120710 28790824 PMC5488784

[28] Eapen V Nicholls L Spagnol V Mathew NE. Current status of biological treatment options in autism spectrum disorder. Asian J Psychiatr. (2017) 30:1–10. 10.1016/j.ajp.2017.07.025 28704714

[29] D'Agati D Chang AD Wachtel LE Reti IM. Treatment of severe self-injurious behavior in autism spectrum disorder by neuromodulation. J ECT. (2017) 33:7–11. 10.1097/YCT.0000000000000346 27428475

[30] Cheng N Rho JM Masino SA. Metabolic dysfunction underlying autism spectrum disorder and potential treatment approaches. Front Mol Neurosci. (2017) 10:34. 10.3389/fnmol.2017.00034 28270747 PMC5318388

[31] Bang M Lee SH Cho SH Yu SA Kim K Lu HY. Herbal medicine treatment for children with autism spectrum disorder: a systematic review. Evid Based Complement Alternat Med. (2017) 2017:8614680. 10.1155/2017/8614680 28592982 PMC5448044

[32] Wink LK Early M Schaefer T Pottenger A Horn P McDougle CJ. Body mass index change in autism spectrum disorders: comparison of treatment with risperidone and aripiprazole. J Child Adolesc Psychopharmacol. (2014) 24:78–82. 10.1089/cap.2013.0099 24564519 PMC5248544

[33] Fung LK Mahajan R Nozzolillo A Bernal P Krasner A Jo B. Pharmacologic treatment of severe irritability and problem behaviors in autism: a systematic review and meta-analysis. Pediatrics. (2016) 137(Suppl 2S):124–35. 10.1542/peds.2015-2851K 26908468

[34] Sahoo S Padhy SK Singla N Singh A. Effectiveness of clozapine for the treatment of psychosis and disruptive behaviour in a child with atypical autism: a case report and a brief review of the evidence. Asian J Psychiatr. (2017) 29:194–5. 10.1016/j.ajp.2017.07.012 28704788

[35] Fitzpatrick SE Srivorakiat L Wink LK Pedapati EV Erickson CA. Aggression in autism spectrum disorder: presentation and treatment options. Neuropsychiatr Dis Treat. (2016) 12:1525–38. 10.2147/NDT.S84585 27382295 PMC4922773

[36] Doyle CA McDougle CJ. Pharmacologic treatments for the behavioral symptoms associated with autism spectrum disorders across the lifespan. Dialogues Clin Neurosci. (2012) 14:263–79. 23226952 10.31887/DCNS.2012.14.3/cdoyle PMC3513681

[37] Hirota T Veenstra-Vanderweele J Hollander E Kishi T. Antiepileptic medications in autism spectrum disorder: a systematic review and meta-analysis. J Autism Dev Disord. (2014) 44:948–57. 10.1007/s10803-013-1952-2 24077782

[38] Antel J Hebebrand J. Weight-reducing side effects of the antiepileptic agents topiramate and zonisamide. In: Joost HG, editor, Appetite Control. Handbook of Experimental Pharmacology, vol 209. Berlin; Heidelberg: Springer. (2012) 433–66. 10.1007/978-3-642-24716-3_20 22249827

[39] Ijff DM Aldenkamp AP. Cognitive side-effects of antiepileptic drugs in children. Handb Clin Neurol. (2013) 111:707–18. 10.1016/B978-0-444-52891-9.00073-7 23622218

[40] Dreifuss FE Langer DH. Side effects of valproate. Am J Med. (1988) 84:34–41. 10.1016/0002-9343(88)90055-1 3146224

[41] Duggal HS Psychotic symptoms associated with topiramate: cognitive side effects or worsening of psychosis? J Clin Psychiatry. (2004) 65:1145 10.4088/JCP.v65n0818c 15323603

[42] Hesami O Hosseini SS Kazemi N Hosseini-Zijoud SM Moghaddam NB Assarzadegan F. Evaluation of ocular side effects in the patients on topiramate therapy for control of migrainous headache. J Clin Diagn Res. (2016) 10:NC01–4. 10.7860/JCDR/2016/16263.7339 27134906 PMC4843292

[43] Nicolai J Smith SJ Keunen RW. Simultaneous side effects of both clozapine and valproate. Intensive Care Med. (2001) 27:943. 10.1007/s001340100937 11430560

[44] Wood JR Nelson-Degrave VL Jansen E McAllister JM Mosselman S Strauss JF 3rd. Valproate-induced alterations in human theca cell gene expression: clues to the association between valproate use and metabolic side effects. Physiol Genomics. (2005) 20:233–43. 10.1152/physiolgenomics.00193.2004 15598877

[45] Koliqi R Polidori C Islami H. Prevalence of side effects treatment with carbamazepine and other antiepileptics in patients with epilepsy. Mater Sociomed. (2015) 27:167–71. 10.5455/msm.2015.27.167-171 26236162 PMC4499297

[46] Langbehn DR Alexander B. Increased risk of side-effects in psychiatric patients treated with clozapine and carbamazepine: a reanalysis. Pharmacopsychiatry. (2000) 33:196. 11071023

[47] Treat L Chapman KE Colborn KL Knupp KG. Duration of use of oral cannabis extract in a cohort of pediatric epilepsy patients. Epilepsia. (2017) 58:123–7. 10.1111/epi.13617 27859038

[48] Slomski A. Fewer seizures with cannabidiol in catastrophic epilepsy. JAMA. (2017) 318:323. 10.1001/jama.2017.8846 28742905

[49] Rosenberg EC Louik J Conway E Devinsky O Friedman D. Quality of life in childhood epilepsy in pediatric patients enrolled in a prospective, open-label clinical study with cannabidiol. Epilepsia. (2017) 58:e96–100. 10.1111/epi.13815 28617940 PMC5568670

[50] Ridler C. Epilepsy: Cannabidiol reduces seizure frequency in Dravet syndrome. Nat Rev Neurol. (2017) 13:383. 10.1038/nrneurol.2017.86 28621765

[51] O'Connell BK Gloss D Devinsky O. Cannabinoids in treatment-resistant epilepsy: a review. Epilepsy Behav. (2017) 70(Pt B):341–8. 10.1016/j.yebeh.2016.11.012 28188044

[52] Devinsky O Cross JH Laux L Marsh E Miller I Nabbout R. Trial of cannabidiol for drug-resistant seizures in the dravet syndrome. N Engl J Med. (2017) 376:2011–20. 10.1056/NEJMoa1611618 28538134

[53] Aguirre-Velazquez CG. Report from a survey of parents regarding the use of cannabidiol (medicinal cannabis) in Mexican children with refractory epilepsy. Neurol Res Int. (2017) 2017:2985729. 10.1155/2017/2985729 28392943 PMC5368357

[54] Tzadok M Uliel-Siboni S Linder I Kramer U Epstein O Menascu S. CBD-enriched medical cannabis for intractable pediatric epilepsy: the current Israeli experience. Seizure. (2016) 3541–44. 10.1016/j.seizure.2016.01.004 26800377

[55] Rosemergy I Adler J Psirides A. Cannabidiol oil in the treatment of super refractory status epilepticus. A case report. Seizure. (2016) 35:56–8. 10.1016/j.seizure.2016.01.009 26803051

[56] Devinsky O Marsh E Friedman D Thiele E Laux L Sullivan J. Cannabidiol in patients with treatment-resistant epilepsy: an open-label interventional trial. Lancet Neurol. (2016) 15:270–8. 10.1016/S1474-4422(15)00379-8 26724101

[57] Press CA Knupp KG Chapman KE. Parental reporting of response to oral cannabis extracts for treatment of refractory epilepsy. Epilepsy Behav. (2015) 45:49–52. 10.1016/j.yebeh.2015.02.043 25845492

[58] Hussain SA Zhou R Jacobson C Weng J Cheng E Lay J. Perceived efficacy of cannabidiol-enriched cannabis extracts for treatment of pediatric epilepsy: a potential role for infantile spasms and Lennox-Gastaut syndrome. Epilepsy Behav. (2015) 47:138–41. 10.1016/j.yebeh.2015.04.009 25935511

[59] Cilio MR Thiele EA Devinsky O. The case for assessing cannabidiol in epilepsy. Epilepsia. (2014) 55:787–90. 10.1111/epi.12635 24854434

[60] Porter BE Jacobson C. Report of a parent survey of cannabidiol-enriched cannabis use in pediatric treatment-resistant epilepsy. Epilepsy Behav. (2013) 29:574–7. 10.1016/j.yebeh.2013.08.037 24237632 PMC4157067

[61] Siniscalco D Sapone A Giordano C Cirillo A de Magistris L Rossi F Cannabinoid receptor type 2, but not type 1, is up-regulated in peripheral blood mononuclear cells of children affected by autistic disorders. J Autism Dev Disord. (2013) 43:2686–95. 10.1007/s10803-013-1824-9 23585028

[62] Karhson DS Krasinska KM Dallaire JA Libove RA Phillips JM Chien AS. Plasma anandamide concentrations are lower in children with autism spectrum disorder. Mol Autism. (2018) 9:18. 10.1186/s13229-018-0203-y 29564080 PMC5848550

[63] Busquets-Garcia A Gomis-Gonzalez M Guegan T Agustin-Pavon C Pastor A Mato S. Targeting the endocannabinoid system in the treatment of fragile X syndrome. Nat Med. (2013) 19:603–7. 10.1038/nm.3127 23542787

[64] Cao X Tabuchi K. Functions of synapse adhesion molecules neurexin/neuroligins and neurodevelopmental disorders. Neurosci Res. (2017) 1163–9. 10.1016/j.neures.2016.09.005 27664583

[65] Jung KM Sepers M Henstridge CM Lassalle O Neuhofer D Martin H. Uncoupling of the endocannabinoid signalling complex in a mouse model of fragile X syndrome. Nat Commun. (2012) 3:1080. 10.1038/ncomms2045 23011134 PMC3657999

[66] Kerr DM Downey L Conboy M Finn DP Roche M. Alterations in the endocannabinoid system in the rat valproic acid model of autism. Behav Brain Res. (2013) 249:124–32. 10.1016/j.bbr.2013.04.043 23643692

[67] Qin M Zeidler Z Moulton K Krych L Xia Z Smith CB. Endocannabinoid-mediated improvement on a test of aversive memory in a mouse model of fragile X syndrome. Behav Brain Res. (2015) 291:164–71. 10.1016/j.bbr.2015.05.003 25979787 PMC5003021

[68] Malcher-Lopes R Cannabinoids help to unravel etiological aspects in common and bring hope for the treatment of autism and epilepsy. Rev Biol. (2014) 13:43–59. 10.7594/revbio.13.01.07

[69] Chez MG Chang M Krasne V Coughlan C Kominsky M Schwartz A. Frequency of epileptiform EEG abnormalities in a sequential screening of autistic patients with no known clinical epilepsy from 1996 to 2005. Epilepsy Behav. (2006) 8:267–71. 10.1016/j.yebeh.2005.11.001 16403678

[70] Markram K Markram H. The intense world theory - a unifying theory of the neurobiology of autism. Front Hum Neurosci. (2010) 4:224. 10.3389/fnhum.2010.00224 21191475 PMC3010743

[71] Shannon S Opila-Lehman J. Effectiveness of cannabidiol oil for pediatric anxiety and insomnia as part of posttraumatic stress disorder: a case report. Perm J. (2016) 20:108–111. 10.7812/TPP/16-005 27768570 PMC5101100

[72] Blessing EM Steenkamp MM Manzanares J Marmar CR. Cannabidiol as a potential treatment for anxiety disorders. Neurotherapeutics. (2015) 12:825–36. 10.1007/s13311-015-0387-1 26341731 PMC4604171

[73] Bergamaschi MM Queiroz RH Chagas MH de Oliveira DC De Martinis BS Kapczinski F. Cannabidiol reduces the anxiety induced by simulated public speaking in treatment-naive social phobia patients. Neuropsychopharmacology. (2011) 36:1219–26. 10.1038/npp.2011.6 21307846 PMC3079847

[74] Zuardi AW Cosme RA Graeff FG Guimaraes FS. Effects of ipsapirone and cannabidiol on human experimental anxiety. J Psychopharmacol. (1993) 7(1 Suppl):82–8. 10.1177/026988119300700112 22290374

[75] Zuardi AW Shirakawa I Finkelfarb E Karniol IG. Action of cannabidiol on the anxiety and other effects produced by delta 9-THC in normal subjects. Psychopharmacology. (1982) 76:245–50. 10.1007/BF00432554 6285406

[76] Zuardi AW Morais SL Guimaraes FS Mechoulam R. Antipsychotic effect of cannabidiol. J Clin Psychiatry. (1995) 56:485–6. 7559378

[77] Zuardi AW Crippa JA Hallak JE Bhattacharyya S Atakan Z Martin-Santos R. A critical review of the antipsychotic effects of cannabidiol: 30 years of a translational investigation. Curr Pharm Des. (2012) 18:5131–40. 10.2174/138161212802884681 22716160

[78] Iseger TA Bossong MG. A systematic review of the antipsychotic properties of cannabidiol in humans. Schizophr Res. (2015) 162:153–61. 10.1016/j.schres.2015.01.033 25667194

[79] Seeman P. Cannabidiol is a partial agonist at dopamine D2High receptors, predicting its antipsychotic clinical dose. Transl Psychiatry. (2016) 6:e920. 10.1038/tp.2016.195 27754480 PMC5315552

[80] Leweke FM Piomelli D Pahlisch F Muhl D Gerth CW Hoyer C. Cannabidiol enhances anandamide signaling and alleviates psychotic symptoms of schizophrenia. Transl Psychiatry. (2012) 2:e94. 10.1038/tp.2012.15 22832859 PMC3316151

[81] Kerr DM Gilmartin A Roche M. Pharmacological inhibition of fatty acid amide hydrolase attenuates social behavioural deficits in male rats prenatally exposed to valproic acid. Pharmacol Res. (2016) 113(Pt A):228–35. 10.1016/j.phrs.2016.08.033 27592249

[82] Lorenz R. On the application of cannabis in paediatrics and epileptology. Neuro Endocrinol Lett. (2004) 25:40–4. 15159680

[83] Jones RM Carberry C Hamo A Lord C. Placebo-like response in absence of treatment in children with Autism. Autism Res. (2017) 10:1567–72. 10.1002/aur.1798 28401674

