# Supplementary material for: Effects of CBD-Enriched Cannabis sativa Extract on Autism Spectrum Disorder Symptoms: An Observational Study of 18 Participants Undergoing Compassionate Use
Source: Front Neurol. 2019 Oct 31;10:1145. doi: 10.3389/fneur.2019.01145 (PMC6834767; doi:10.3389/fneur.2019.01145)
Supplement: Supplementary file 1 [file Data_Sheet_1.PDF]

Supplementary Material

Effects of CBD-enriched *Cannabis sativa* extract on Autism Spectrum Disorder symptoms: an observational study of 18 subjects undergoing compassionate use

Paulo Fleury-Teixeira, Fabio Viegas Caixeta, Leandro Cruz Ramires da Silva, Joaquim Pereira Brasil-Neto and Renato Malcher-Lopes*

*** Correspondence:** Renato Malcher-Lopes**: malcherlopes@gmail.com**

PATIENT'S PERSONAL HEALTH DATA (Translated from Brazilian Portuguese)

GUIDANCE FOR COMPLETING THE PATIENT'S PERSONAL HEALTH DATA

DURING THE EXPERIMENTAL TREATMENT:

Fill in the information below as accurately as you can.

In the Time Period area inform the month of treatment.

When describing the main characteristics in the period (in each dimension of signs and symptoms that relate to the autistic spectrum disorders presented by the patient), inform the main characteristics of the patient in each aspect of that dimension. In the personal health data records of progression inform the observed changes in the period, for each aspect under consideration. If the patient does not have the signs and symptoms in question, write: *"None"*. If there has been no significant change in the period, write: *"There was no change."*

PATIENT'S NAME:

Time Period:

Weight:

1. Hyperactivity and attention deficits - Frequency and intensity of restlessness and excessive movement during daily activities. Lack of focus. Difficulty in staying seated or in the same place when performing tasks.

Main characteristics in the period: (in this section the caretaker has the opportunity to inform in his/her own words the signs/symptoms presented by the patient during the previous month)

On a scale from 0 to 100 (with zero being maximum hyperactivity and attention deficit, and one hundred the ideal condition):

Before starting the oil treatment:

Current:

Perception of Monthly Improvement:

( ) Acute worsening

( ) Worsening

( ) Stability

( ) Improvement

( ) Sharp improvement

2. Stereotypies and abnormal behavior - Episodes of aggression, self-aggressiveness, repetitive behaviors, binge eating, excessive fear reactions, panic, crying or laughter spells, and other aberrant behaviors. Flapping and other repetitive movements.

Main characteristics in the period: (in this section the caretaker has the opportunity to inform in his/her own words the signs/symptoms presented by the patient during the previous month)

On a scale from 0 to 100 (with zero being the maximum of stereotypies and aberrant behaviors, and one hundred ideal condition):

Before starting the oil treatment:

Current:

Perception of Monthly Improvement:

( ) Acute worsening

( ) Worsening

( ) Stability

( ) Improvement

( ) Sharp improvement

3. Motor development - General motor coordination, walking and physical activities in general. Fine motor coordination, manual dexterity.

Main characteristics in the period: (in this section the caretaker has the opportunity to inform in his/her own words the signs/symptoms presented by the patient during the previous month)

On a scale from 0 to 100 (with zero being the worst case scenario and one hundred the ideal condition):

Before starting the oil treatment:

Current:

Perception of Monthly Improvement:

( ) Acute worsening

( ) Worsening

( ) Stability

( ) Improvement

( ) Sharp improvement

4. Functional dependence for daily activities - Degree of dependency regarding feeding, dressing, bathroom needs and personal hygiene (using the bathroom, using the toilet, etc.), moving around and when performing other activities.

Main characteristics in the period: (in this section the caretaker has the opportunity to inform in his/her own words the signs/symptoms presented by the patient during the previous month)

On a scale from 0 to 100 (with zero being total incapacity and one hundred the ideal condition):

Before starting the oil treatment:

Current:

Perception of Monthly Improvement:

( ) Acute worsening

( ) Worsening

( ) Stability

( ) Improvement

( ) Sharp improvement

5. Verbal and non-verbal communication and interaction - Expression and understanding in the various forms of communication. Visual contact and social interaction (family, school, friendship, social networks).

Main characteristics in the period: (in this section the caretaker has the opportunity to inform in his/her own words the signs/symptoms presented by the patient during the previous month)

On a scale from 0 to 100 (with zero being total incapacity and one hundred the ideal condition):

Before starting the oil treatment:

Current:

Perception of Monthly Improvement:

( ) Acute worsening

( ) Worsening

( ) Stability

( ) Improvement

( ) Sharp improvement

6. Cognitive performance - Learning and development in reading / writing / math / technology / arts etc.

Main characteristics in the period: (in this section the caretaker has the opportunity to inform in his/her own words the signs/symptoms presented by the patient during the previous month)

On a scale from 0 to 100 (with zero being total incapacity and one hundred the ideal condition):

Before starting the oil treatment:

Current:

Perception of Monthly Improvement:

( ) Acute worsening

( ) Worsening

( ) Stability

( ) Improvement

( ) Sharp improvement

7. Sleep Disorders - Insomnia, difficulty to sleep, intense nocturnal agitation, awakenings. Inform frequency and intensity of episodes.

Main characteristics in the period: (in this section the caretaker has the opportunity to inform in his/her own words the signs/symptoms presented by the patient during the previous month)

On a scale from 0 to 100 (with zero being the worst case scenario and one hundred the ideal condition):

Before starting the oil treatment:

Current:

Perception of Monthly Improvement:

( ) Acute worsening

( ) Worsening

( ) Stability

( ) Improvement

( ) Sharp improvement

8. Epileptic seizures - Inform average frequency per day, week, fortnight or month. Inform type and duration of crises.

Main characteristics in the period: (in this section the caretaker has the opportunity to inform in his/her own words the signs/symptoms presented by the patient during the previous month)

Perception of Monthly Improvement:

( ) Acute worsening

( ) Worsening

( ) Stability

( ) Improvement

( ) Sharp improvement

9. Inform the presence of **adverse side effects attributable to Cannabis oil in the period:**

10. Other medications for ASD / Sleep Disorder / Epilepsy / Psychiatric conditions. Inform any alterations of other medications currently in use / Introduction of new medication / Discontinuation of medication use / Change of dosage / Substitution of medication / Adverse side effects.
